# Preclinical Evaluation of CAR T Cell Function: In Vitro and In Vivo Models

**DOI:** 10.3390/ijms23063154

**Published:** 2022-03-15

**Authors:** Xiaohui Si, Lu Xiao, Christine E. Brown, Dongrui Wang

**Affiliations:** 1 Bone Marrow Transplantation Center of the First Affiliated Hospital, Zhejiang University School of Medicine, Hangzhou 310003, China; xiaohui523@zju.edu.cn; 2 Liangzhu Laboratory, Zhejiang University Medical Center, 1369 West Wenyi Road, Hangzhou 311121, China; 3 Institute of Hematology, Zhejiang University, Hangzhou 310030, China; 4 Zhejiang Province Engineering Laboratory for Stem Cell and Immunity Therapy, Hangzhou 310030, China; 5 Canyon Vista Medical Center, Midwestern University Consortium, Sierra Vista, AZ 85635, USA; luxiaolindaus@gmail.com; 6 Department of Hematology and Hematopoietic Cell Transplantation, City of Hope Medical Center, Duarte, CA 91010, USA; cbrown@coh.org

**Keywords:** adoptive cell transfer, antitumor immunity, tumor microenvironment

## Abstract

Immunotherapy using chimeric antigen receptor (CAR) T cells is a rapidly emerging modality that engineers T cells to redirect tumor-specific cytotoxicity. CAR T cells have been well characterized for their efficacy against B cell malignancies, and rigorously studied in other types of tumors. Preclinical evaluation of CAR T cell function, including direct tumor killing, cytokine production, and memory responses, is crucial to the development and optimization of CAR T cell therapies. Such comprehensive examinations are usually performed in different types of models. Model establishment should focus on key challenges in the clinical setting and the capability to generate reliable data to indicate CAR T cell therapeutic potency in the clinic. Further, modeling the interaction between CAR T cells and tumor microenvironment provides additional insight for the future endeavors to enhance efficacy, especially against solid tumors. This review will summarize both in vitro and in vivo models for CAR T cell functional evaluation, including how they have evolved with the needs of CAR T cell research, the information they can provide for preclinical assessment of CAR T cell products, and recent technology advances to test CAR T cells in more clinically relevant models.

## 1. Introduction

The immune system plays an essential role in the initiation, progression, and metastasis of cancer [1,2]. Meanwhile, immune cells can also be utilized as cancer-targeting agents [3,4]. Adoptive transfer of T cells engineered with chimeric antigen receptors (CARs) has resulted in tremendous breakthroughs in cancer therapy against multiple types of tumors [4,5,6,7]. CAR is a synthetic immune receptor that consists of an extracellular tumor-binding domain (derived from antibodies, ligands, or peptides) that recognizes tumor-associated antigens (TAAs), together with intracellular T cell activation signals (CD3 and co-stimulation domains) [8]. CAR engineering allows T cells to bypass the HLA restriction for tumor recognition, and its modular design makes it convenient for modification against different TAAs [9].

CAR T cell therapy has seen the most promising clinical responses against tumors of the B cell lineages [10,11,12], which also led to the first ever approved cellular therapy for cancer and first ever approved gene therapy by the U.S. Food and Drug Administration (FDA) [13]. Thus far, five CAR T cell therapies have gone through FDA approval, targeting B cell leukemia [13,14], lymphoma [15,16], and multiple myeloma [17]. CAR T cells for solid tumors, however, have resulted in mixed and inconsistent clinical outcomes [18], but some clinical evidence has suggested the induction of antitumor effect mediated by CAR and endogenous T cells [19,20,21,22], including a patient with refractory multifocal glioblastomas that achieved a complete response after CAR T cell therapy [19]. Further, tumor relapse may occur in patients who initially responded well to CAR T cells [23,24]. Antigen loss/escape and CAR T cell dysfunction are two major hurdles against effective CAR T cell therapy [25,26], directing future investigation on CAR T cell refinement, which includes the modification of CAR constructs, optimization of manufacturing and designs of clinical studies [3,4,9,27].

The intrinsic quality and fitness of CAR T cells are closely associated with clinical outcomes [28,29], illustrating the need for comprehensive preclinical examinations on the cellular products. Given the fact that current CAR optimization is still partially empirical based on the targeting of different TAAs, utilizing appropriate models to evaluate CAR T cell function is crucial for acquiring reliable results that inform clinical development. In this review, we discuss the models used to preclinically evaluate CAR T cells, focused on the advantages and limitations of each model for specific parameters of CAR T cell function.

## 2. How CAR T Cells Should Be Evaluated: Critical Parameters

**Figure 1:**
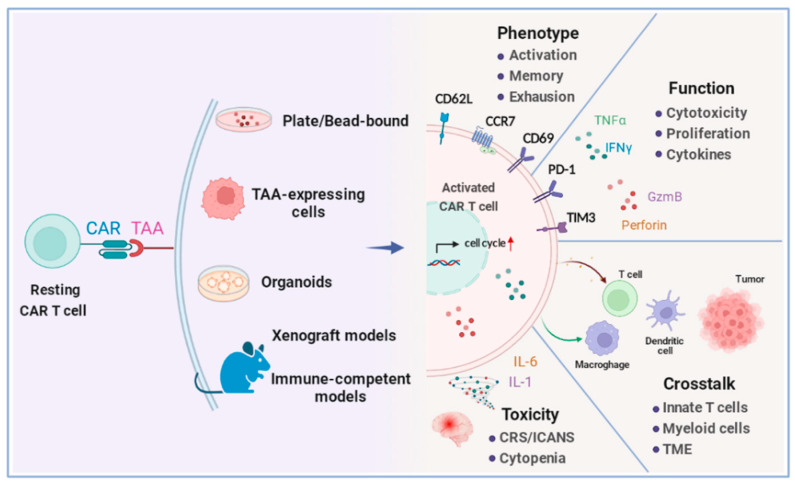
Schematic of functional parameters on activated CAR T cells.

For clinical-grade CAR T cell products, regular quality controls are required before each infusion, which usually include sterility, cell viability, purity (cleanliness of bead removal), identity (T cell percentage), CAR expression, and potency [30,31]. CAR T functional evaluation in preclinical studies can be more complex, since these products need to be rigorously tested before entering clinical development. To make the assessment as accurate as possible, these examinations should cover a wide range of CAR T cell function-related factors (Figure 1).

Resting CAR T cells are activated upon CAR recognition of tumor-associated antigens (TAAs), which can be coated onto a culture plate (plate-bound)/nanobead (bead-bound), or expressed on target cells in different models. Activated CAR T cells are able to proliferate/expand, produce cytotoxic molecules, such as granzyme (GzmB) and perforin, and secrete different types of cytokines. Surface markers are exploited to indicate the developmental and functional stages of CAR T cells, including activation (CD69+), memory (CCR7+, CD62L+), and exhaustion (PD-1+, TIM-3+). CAR T cells also mediate crosstalk with the host immune system and tumor microenvironment (TME), and may cause adverse effects, such as CRS, ICANS, or cytopenia, which have to be carefully evaluated and monitored.

First, CAR T cells eliminate tumor cells via direct cytotoxicity, which can be tested by the capability to degranulate (expression of CD107a), to produce cytotoxic molecules (i.e., granzyme B and perforin), and to induce cell death in target/tumor cells [32]. Meanwhile, activated CAR T cells have the potential to proliferate and expand, which has been observed in CAR-treated patients [10,11]. Killing and expansion potency is always the first line of CAR T cell functional evaluation.

When CAR T cells are activated, they start to produce various types of cytokines. Some of these cytokines (such as IL-2, IL-15, IFN-γ, and TNF-α) can facilitate CAR T expansion and killing, along with the activation of other immune populations, while others (such as IL-6, IL-10, and TGF-β) inhibit antitumor immune function [33]. Further, cytokine release syndrome (CRS) is frequently observed in patients treated with CAR T cell therapy and sometimes leads to severe adverse effects [34,35,36]. It is, thus, critical to understand the cytokine profile of CAR T cells (both types and amounts of cytokine secreted) as important information of both efficacy and toxicity.

T cell surface markers can undergo dynamic changes during their differentiation, activation, and memory formation. These surface markers are, therefore, exploited to indicate the developmental and functional stages of T cells. Studies have found that surface marker profiles of CAR T cells can be associated with clinical responses. Generally, CAR T cells with a memory-like phenotype (expression of surface markers CD62L, CCR7, CD45RA, and CD45RO) can mediate superior efficacy [28,37,38], while the acquisition of exhaustion-like phenotype (expression of inhibitory receptors, such as PD-1, LAG-3, and TIM-3) in CAR T cells limits their potency [39,40,41]. It has also been revealed that CD4+ CAR T cells can mediate a more potent antitumor response than CD8+ cells against some solid tumors [42,43]. Analyzing these markers on CAR T cells, in parallel to other functional tests, would provide a comprehensive understanding of the underlying biology for how CAR T cells maintain or lose their antitumor function.

T cells are an essential component of the adaptive immune response, regulating many other types of immune cells to target infection and tumors [44,45,46]. Conceivably, the activation of CAR T cells, and the subsequent cytokine production, may impact or even reprogram tumor cells and the tumor microenvironment (TME) [47]. Clinical research has revealed that the endogenous immune response can be potentiated after CAR T cell therapy and may contribute to antitumor efficacy [19,20]. Therefore, the analyses of other types of immune cells and how they can be impacted by CAR T cell therapy would greatly contribute to the preclinical evaluation of CAR T cells.

One of the main barriers against wider CAR T cell clinical application is its toxicity. Toxicity can result from CAR T cells targeting nontumor cells expressing different levels of TAAs (on-target, off-tumor), or by the activation of CAR T cells and the subsequent induction of systemic immunity [48]. Toxicity has been presented with various symptoms, illustrated by CRS and immune effector cell-associated neurotoxicity syndrome (ICANS) [49]. Preclinical evaluation of CAR T cell toxicity would require surrogate models, especially human-derived models, that recapitulate the clinical manifestations of toxicity-related events.

## 3. In Vitro Models for CAR T Cell Functional Evaluation

### 3.1. TAA-Dependent Cytotoxicity Assessment

CAR T cell function can be evaluated in vitro by examining effector responses against targeted TAAs. Recombinant TAAs can be either coated onto a culture plate (plate-bound) or a nanobead (bead-bound). An alternative approach is Protein L, which activates most antibody-based CARs and has been used to track the internalization of CAR molecules [50]. The TAAs can also be endogenously expressed or engineered to be expressed on target cell lines. Plate-bound TAAs only provide activation through CARs, while TAA-expressing cells may have additional interaction with CAR T cells through co-stimulation engagement, adhesion molecule interactions, and immune-suppressive ligands. If the studies aim to compare several CAR T cell products targeting different TAAs, it is then suggested that a fixed type of target cell be engineered to express these TAAs at similar densities. The leukemia cell line Nalm-6 has been particularly recommended as the parental cells, given its lack of costimulatory ligands, which reduces the noise when assessing CAR activation [39,51].

When CAR T cells are analyzed using these in vitro models, researchers have easy access to the samples for most of the required parameters listed above: the supernatant of the culture can be harvested to assess cytokine production; the target cells can be harvested for flow cytometric analyses to evaluate killing potency (by counting the remaining viable target cells), and CAR T cell proliferation and phenotype. Recently developed technologies have allowed for the analyses of single-cell cytokine production in a TAA-expressing cell coculture model, leading to the assessment of CAR T cell polyfunctionality [52,53,54]. Polyfunctional CAR T cells were featured by their capability to simultaneously secrete multiple types of immune-stimulatory cytokines and cytotoxic molecules at a single-cell level [55,56,57], which can serve as a biomarker of clinical outcomes of CD19-CAR T cells [55]. A similar strategy can also be applied to measure immune-suppressive cytokines on tumor cells, supporting the investigations of adaptive resistance to immunotherapy [58].

In vitro models are relatively less labor-intensive and easier to scale up, making them particularly favorable for studies of CAR T cell activation-related signaling pathways, which require a large number of cells harvested for biochemical assays [59,60]. These systems also provide a rapid method, serving as proof-of-concept assays to evaluate combinational treatment of CAR T cells with small-molecule drugs, such as the inhibitors of tyrosine kinases (i.e., Akt and Src) [61,62], EZH [63], ITAM [60], calcium signaling [64], and ubiquitination [65]. Further, as TAA-expressing cells grow rapidly, they can be clonally sorted and expanded to evaluate CAR T cell activation potential against different TAA densities that represent clinical settings [50,66].

While these in vitro models are indeed artificial mixtures of CAR T cells and their targets, they can still be modified to approximate the high tumor burden seen in actual clinical applications. Through sequential plating (when using precoated TAAs) [41], lowering effector:target (E:T) ratios and applying rechallenge (when using TAA-expressing cells) [43,67,68,69], researchers are able to recreate the condition where CAR T cells are repetitively exposed to TAAs, a process known to mediate T cell loss of function [70]. These more challenging in vitro models can support CAR T cell designs that resist exhaustion. Further, with the technical advances of real-time imaging [71], CAR T cell cytotoxicity can be monitored overtime at varying E:T ratios, allowing for mathematical modeling and more precise evaluation of killing kinetics [72].

### 3.2. Tumor-Derived Organoids for CAR T Cell Therapy

Recombinant TAA and cell-line-based models are unable to recapitulate the heterogenous nature of most solid tumors, which has become the major hurdle for in vitro modeling systems to represent the clinical reality. Organoids are three-dimensional, multi-cellular structures that resemble the spatial characteristics of the original tissue [73,74]. Tumor-derived organoids (TDOs) have been initially generated from normal organoids by introducing pro-tumorigenic mutations, such as the losses of APC and TP53 [75,76], and then directly from fresh tumor specimens, which capture the cellular and molecular diversity of the clinical samples [77,78,79,80,81,82].

Jacob et al. used brain tumor TDOs to demonstrate that EGFRvIII-targeted CAR T cells can infiltrate and elicit tumor cell apoptosis in this model [83], suggesting the feasibility of TDOs in preclinical CAR T cell evaluation. The heterogenous TDOs also recapitulated antigen escape, as only EGFRvIII-expressing cells were eliminated after applying CAR T cells [83]. Another study tested FRIZZLED-targeted CAR-NK cells against colon cancer TDOs versus organoids from normal gastric tissue, which revealed a lack of tumor specificity of these CAR-NK cells [84]. These studies have suggested the potential of TDOs as a more clinically relevant in vitro model system, especially for heterogeneous solid tumors. Further, bioreactors have been applied for large-scale generation of brain organoids, which can be extended to TDOs for CAR functional evaluation [85]. Intriguingly, HER2-targeted CAR T cells were found to kill colon cancer TDOs in a perforin-independent manner mediated by TNF [86], indicating that CAR T cell cytotoxicity might require distinct mechanisms against TDOs versus two-dimensional cell culture. Future research needs to focus on the comparison of CAR-mediated T cell biological processes (activation, memory formation, exhaustion) between TDOs and other in vitro models.

Early-stage TDOs only consisted of tumor cells, but recent efforts have been focused on utilizing TDOs to restore or reconstitute the TME [74,87], thus providing more information beyond considerations of tumor heterogeneity. For example, tumor-reactive T cells can be detected when lung or colorectal cancer TDOs were cocultured with peripheral blood mononuclear cells (PBMCs) [88], suggesting that TDOs can be exploited to discover new tumor antigens. Meanwhile, TDOs allow for the investigation of endogenous CD8+ T cell infiltrates that may have synergistic effect with CAR-mediated tumor killing [88,89]. Another direction includes the studies of vascularization in TDOs, which can be achieved by adding endothelial cells [90] or mesodermal progenitor cells [89], thereby providing mechanistic insights about how CAR T cells penetrate tumor stroma. Developing these complex TDOs will benefit the future in vitro modeling of CAR T cell functional evaluation.

## 4. In Vivo Models for CAR T Cell Functional Evaluation

Clinical challenges of CAR T cell therapy have revealed the need to understand more detailed cellular mechanisms in preclinical investigations, which requires multi-cellular modeling systems consisting of both tumor and other types of cells in the TME [91]. These models are usually established in vivo with human or mouse tumors. While the antitumor efficacy of CAR T cells is typically the primary readout of these models, researchers can also acquire other important information, including CAR T cell persistence, cytokine production, and therapy-related alterations in the TME. Analyses of TME can be achieved by examining post-treatment tumors and other surrogate samples, such as cerebral spinal fluid for brain tumors [92,93] and ascites for intraperitoneal tumors [94]. Depending on the model(s) used, in vivo studies enable: (i) examining CAR T cell function in established large tumors; (ii) monitoring the dynamics of antitumor effect throughout the course of treatment; (iii) investigating the crosstalk between CAR T cells and the host immune system; (iv) assessing the trafficking of systemically injected cells into solid tumors; and (v) evaluating therapy-related toxicity.

Below, we discuss two major types of in vivo models: immune-compromised models and immune-competent models. They both provide informative platforms to evaluate CAR T cells in a more complex, reliable, and realistic environment, and should be appropriately utilized depending on the scientific questions to be addressed.

### 4.1. Immune-Compromised (Xenograft) Models

Mice with deficient immune systems enable the engraftment of human tumors (xenografts), serving as critical models for CAR T cell evaluation [95]. Most in vivo CAR T cell studies have used the *NOD.Cg-Prkdc^scid^Il2rg^tm1Wjl^/SzJ (NSG) mouse model* [96], with no functional T, B, NK, and dendritic cells, and others used *athymic nude mice* with defects in T cell development [97]. Some models have additional expression of immune-stimulatory cytokine, such as IL-2, to enhance the function of infused human T cells [98].

Tumors can be established using human tumor cell lines or tumor samples from the clinic. Tumor cells/samples of hematopoietic origin are usually injected systemically (intravenous or intracardiac injections). Solid tumors can be established by inoculating subcutaneously (for easy harvest of tumor tissues), orthotopically (for a more realistic environment of tumor engraftment), or systemically (to mimic tumor invasion and metastasis). Cell lines can be rapidly expanded to generate large numbers of xenografts that enable high-throughput in vivo tests. Clinical tumor samples, which generate patient-derived xenografts (PDXs), are valuable models to recapitulate the genetic and cellular profiles of the original tumors [99,100]. Availability of PDX models, however, is often limited due to insufficient access to primary tumor specimens. Tissues from certain types of tumors, such as glioblastomas, can be expanded in vitro as tumorspheres, while maintaining their molecular signature and stemness before implantation [101,102,103]. TDOs are also a critical resource for establishing PDXs, as heterogenous tumor cells with spatial characterization of the original sample can be expanded in vitro before being transplanted to establish xenografts [104].

Evaluating the in vivo function in xenograft models is an indispensable step in a series of preclinical studies for CAR T cell refinement and optimization, including optimization of CAR designs [66,105,106,107,108,109,110], CAR T cell transduction methods, and manufacturing platforms [38,40,68,111]. In vivo antitumor activity against xenografts has been one of the most critical factors for deciding whether certain CAR designs can be proceeded for clinical development. To get reliable readouts to mimic clinical settings, xenografts are often treated with suboptimal doses of CAR T cells (“CAR stress test”), and tumor rechallenge can be applied to evaluate CAR-mediated persistence and memory responses [40,63,112]. Some invasive and multi-focal tumor models also enable researchers to monitor CAR T cell trafficking [113,114,115]. If CAR T cell targets are conserved between humans and mice, these models can serve as surrogates to assess off-tumor targeting and toxicities of human CAR T cells and provide critical information for clinical development [109,116]. Practically, after CAR T cell therapy, organs (including tumor-bearing organs and other normal organs) are harvested, paraffin-embedded, and sectioned. These sections can be stained to examine any pathological alterations and CAR T cell infiltration. In the scenarios of tumor relapse post CAR T cell therapy, samples need to be analyzed to determine whether the recurrent tumor is a result of antigen escape and/or CAR T cell dysfunction [43,106,107,117].

Of note, immune-compromised mice have not been depleted for all immune cells, making it possible to investigate the endogenous immune response to some extent. In nude mice, M1 macrophages and myeloid-derived suppressor cells (MDSCs) can expand after immune stimulation using LPS or tumors [118], suggesting tumor-induced immunity mediated by the myeloid compartments. In some more immunocompromised models, such as SCID-beige or NSG, studies have also demonstrated the existence of MDSCs and tumor-associated macrophages [92,119]. Myeloid cells in SCID-beige mice can be induced to produce IL-1, IL-6, and nitric oxide after CAR infusions, leading to tumor-unrelated activity reduction, malaise, piloerection, weight loss, and mortality in CAR-treated animals, serving as a CRS model [120]. Meanwhile, targeting MDSCs in NSG mice was able to enhance CAR T cell antitumor function [119]. However, it is acknowledged that the immune components in these models are not sufficient to fully understand the impact of CAR T cell therapy on endogenous antitumor immunity.

### 4.2. Immune-Competent Models

Immune-compromised models fail to recapitulate the intact immune system, which plays an essential role in regulating CAR T cell function. Furthermore, there has been clinical evidence that a crosstalk exists between the infused CAR T cells and the endogenous immune system [19,121,122], suggesting the need to analyze CAR T cells in an immune-competent environment. These models are also critical for mimicking preconditioning regimens, such as lymphodepletion with radiation or chemotherapy [123,124], as well as evaluating immunotherapy approaches combining CAR T cells with immune checkpoint blockade (ICB) [125], oncolytic viruses [126,127], cytokine delivery [128], or agents targeting immune-suppressive signals in the TME [129,130]. Here, we discuss three major types of immune-competent models applied to CAR T cell research.

*Implantable tumor models* are the most widely used immune-competent models in preclinical studies of cancer immunotherapy. These models are established by injecting histo-compatible (syngeneic) tumor cells into immune-competent hosts, which are most commonly BALB/c and C57BL/6 mouse strains [131]. Similar to establishing tumors in PDX models, tumor cells of the hematological system are usually injected systemically, while solid tumor cells are inoculated subcutaneously, orthotopically, or systemically. Orthotopic implantation of solid tumors is thought to better recreate the TME of established tumors [131,132]. Our study in 2021 used orthotopic brain tumor models (KR154 and GL261 cells) to recapitulate the invasive and immune-suppressive features of human gliomas [133]. This study also provided an insightful strategy to evaluate CAR-induced endogenous antitumor response, with initial implantation of tumor cells engineered to express CAR-targeted TAAs, followed by tumor rechallenge using the parental TAA-negative tumor cells [133]. The ability of mice to eradicate TAA-negative tumor cells was a clear indicator of CAR-induced antigen spread and the activation of recipient antitumor immune responses.

CAR-induced host immunity, including antitumor responses of endogenous T cells, activation of NK cells, and polarization of TAMs towards a M1 phenotype, was found in multiple CAR T cell platforms, such as CD19-, IL13Rα2-, VEGFR-, and mesothelin-targeted CARs using implantable syngeneic tumor models [126,133,134,135]. These models are also extremely useful for evaluating “armored” CAR designs, with engineered cytokine or co-stimulation on top of CAR expression. These molecules, such as IL-12 [136], IL-15 [135], IL-18 [137,138], IL-23 [139], and CD40L [140], all mediate the interaction between CAR T cells and the endogenous immune system. Other studies used these models to investigate immune-suppressive pathways, including transforming growth factor beta (TGFβ) signaling, adenosine metabolic pathways, and immune checkpoints [141,142,143,144]. Inhibiting the suppressive signals in immune-competent models enables researchers to investigate the enhanced antitumor immunity from both infused CAR T cells and endogenous T cells. One limitation of implantable tumor models, however, is their artificial nature, which is particularly difficult in studying the development of endogenous antitumor T cells in early-stage tumors.

*Genetically engineered mouse models (GEMMs)* have the potential to overcome these shortcomings, providing a unique system of in situ tumor growth, which can be used to study how antitumor T cells are co-evolved with tumor progression [131]. Tumor GEMMs were established through genetically controlled expression of oncogenes or inactivation of tumor-suppressor genes. Some of these genes, such as *ERBB2* or *EGFR*, have also been used as CAR T cell targets [22,121], suggesting that GEMMs can be informative, especially for CAR T cell activity against early-stage tumors. GEMMs have been used to study endogenous antitumor T cells, and revealed a sequential T cell exhaustion program upon persistent antigen exposure starting early during tumor initiation [145]. Further, antitumor T cells in GEMMs undergo epigenetic reprograming in late-stage tumors, which cannot be reversed by ICB [146]. These discoveries have shed light on the in vivo tumor-killing and exhaustion mechanisms of CAR T cells, and also suggested the necessity to evaluate CAR T endogenous immunity interaction in tumor GEMMs.

One important nontumor GEMM model for CAR T cell studies has been the mice with depletion of recombination activating gene 2 (Rag2-/-), which cannot generate T and B cells [147]. This model has been used to demonstrate the impact of endogenous lymphocytes on CAR T cell antitumor effect [123]. Additionally, a recent study used a GEMM model with a knock-in of CAR-encoded sequences to generate EGFRvIII-targeted CAR T cells in vivo, and showed effector function against implanted syngeneic tumors and can be an alternative approach to test early-stage CAR antitumor responses [148].

*Humanized mouse (HM) models* have been developed and applied to CAR T cell therapy to address the need for modeling human-originated cancers in the presence of intact immune systems [149]. The reconstitution of the immune system is achieved by administering human PBMCs (*periphery blood lymphocytes (PBL-HM) model*) [150] or hematopoietic stem cells (*HSC-HM model*) [151] into immune-deficient mice. Another approach includes the implantation of fetal thymus, fetal liver, and fetal liver HSCs (*BLT-HM model*), which favors the development of human lymphocytes [152]. The recipients are sometimes engineered with MHC molecules (HLA-I/II), human cytokines (SCF, M-CSF, IL-3, GM-CSF, thrombopoietin), or phagocytosis-inhibiting signal (SIRPa) to enhance the engraftment of human hematopoietic cells (strain names of NSG/NRG-SGM3, MITRG, MISTRG) [149,153,154,155]. The establishment of HM models has been greatly supported by the technical advances to expand human HSCs in vitro [156], and to generate HSCs from induced pluripotent stem cells [157].

These models have been used to evaluate CAR T cell function and adverse events. Jin et al. generated a BLT-HM model, with MLL-AF9-transgenic HSCs to develop B-cell leukemia in vivo [158]. CD19-CAR T cell infusion in this model resulted in tumor regression, together with induction of TAM-secreted cytokines and Treg activity, which resembles clinical observations [158]. A similar BLT-HM model without introduction of oncogene was used to evaluate the antiviral function of HIV-targeted CAR T cells [159]. Additionally, CARs targeting one, two, or three HIV epitopes were examined in a PBL-HM model, revealing that multi-specific CARs are required for anti-HIV therapy [160]. Another major direction of HM models has been the evaluation of CAR-induced toxicity. An early study used HSC-HM models to assess the off-target potential, comparing the killing effect of CD33/CD123 CAR T cells against leukemia cells versus normal stem cells [161]. More recently, CRS has been closely investigated in HSC-HM models. One study used HSC-HM models to mimic the cytokine profiles after CAR T cell therapy, and the researchers incorporated an induced apoptosis signal into the CAR construct to achieve CAR depletion and were able to ameliorate the CRS-like symptoms [162]. A separate study used HSC-HM models to identify that CRS and ICANS are highly dependent on IL-1 and IL-6 from the recipient myeloid compartment. Blocking IL-6 was shown to prevent CRS, while blocking IL-1 protected animals from both CRS and ICANS. [163]. These findings are important to managing adverse events in the clinical applications of CAR T cells.

HM models may develop severe graft-versus-host disease (GvHD) [164], which significantly restricts the time window that CAR T cell evaluation can be performed. Peripheral T cells are believed to be the main contributor to GvHD, in line with the rapid GvHD onset in PBL-HM models [165]. Both HSC-HM and BLT-HM models are able to delay GvHD onset, but the latter may still not be widely applicable due to ethical issues. Indeed, HSC-HM have been the commonly used HM models for CAR T cell studies. HM models are also, overall, more time-consuming than other in vivo models due to the additional procedures of hematopoietic cell/tissue engraftment. An ideal “avatar” model requires the implantation of PDXs, together with their autologous human immune cells [166], which is further limited by the low availability of these matched tissues in the clinic. However, HM models have the advantage that they allow for the evaluation of the human CAR T cell product intended for clinical translation. Overcoming these hurdles can make HM models more accessible to serve as treatment guides for CAR T cell therapy in the future.

## 5. Models for CRISPR Screenings in CAR T Cells

Screenings using the CRISPR technology generate loss-of-function or gain-of-function libraries to identify key factors regulating certain biological processes in an unbiased manner [167,168]. This platform has been applied to both tumor cells and immune cells [169]. Screening on tumor cells is less technically challenging and has been used to find genes contributing resistance to many types of immunotherapies, including CAR T cells [170,171,172,173]. One study by Singh et al. further investigated apoptosis pathways in leukemia cells, which were the highest-scored targets in the screen [173]. Inhibiting apoptosis pathways in leukemia cells was shown to disrupt their sensitivity to CAR-mediated cytotoxicity, which subsequently triggered CAR T cell exhaustion due to excessive tumor exposure [173]. Screenings have also been performed on TDOs for tumor-growth dependencies [174], which can be further extended to TDO-CAR coculture models in the future.

Screenings on immune cells, such as T cells, provide a direct approach to identify genes impacting immune responses, with the potential of dictating future cellular immunotherapies [169,175]. Model development is one of the most technically challenging procedures of these screenings. A good balance should be considered between the large numbers of cells needed to cover the screened targets (especially for genome-wide screenings) and the requirement to recapitulate the physiological environment of these cells. In vitro systems are usually easier to scale up and, therefore, are more likely to enable the harvesting of the required cells for adequate library coverage. Early studies have established a CRISPR screening system in human T cells following in vitro TCR stimulation, identifying cell-cycle-related factors essential for T cell activation and proliferation [176]. Our group then developed an in vitro screening platform for human CAR T cells using a tumor cell rechallenge model, followed by CAR T cell sorting based on the surface marker PD-1 [177]. This approach enabled a rigorous comparison between the exhausted versus non-exhausted CAR T cells. Essential genes that prohibit CAR T cell effector function were identified, including TLE4 and IKZF2, which were then validated as targets to ameliorate CAR T cell in vivo antitumor function with high tumor burden models [177].

Technical advances, such as the Cas9-knockin mice, have enabled large-scale generation of mouse T cells harboring a CRISPR library [178]. Combined with mouse viral infection or vaccination models, this screening system has led to a series of in vivo studies for T cell memory, exhaustion, and metabolic regulation [179,180,181]. The identified targets may be exploited to improve the fitness of CAR T cells. In vivo screening against tumor models, however, can be particularly complicated. Tumor-infiltrating T cells are usually the cell populations analyzed, and these cells are mixtures of subsets representing differential states of tumor recognition, trafficking exhaustion, which is further confounded by the inconsistency across tumor models [182,183]. Together with the considerations of inter-species variation between mouse and human, targets identified in these screenings would need extensive validation to elucidate their roles in CAR T cell antitumor activity. For example, an in vivo screening on systemically administered mouse CAR T cells has found some targets enriched in the CAR T cells that trafficked to brain tumors, and further evaluation was performed to show that these targets can enhance cytolytic function in human CAR T cells [183]. A similar strategy of validating targets will be required until better models for in vivo screening on CAR T cells are developed. Alternatively, screenings on more customized, smaller-scale targets can be performed in multiple models and increase the reliability. The study by Roth et al. combined both in vitro and in vivo approaches to screen 36 CRISPR knock-ins of known T cell function-related targets in NY-ESO-1 TCR T cells, identifying a TGFβR2-41BB chimeric receptor that enhanced antitumor function [184]. Such a rapidly integrated library and comprehensive utilization of models is informative to the design of screening strategies on CAR T cells.

Overall, the models for CRISPR screenings will need to be varied across different cell types being screened. To enhance the reliability of these screens, researchers have to adopt challenging models for their cells of interest. For screenings on CAR T cells, the recapitulation of high tumor burden can be achieved by in vitro tumor cell rechallenge at low E:T ratios, or a reliable in vivo tumor model; for screening on tumor cells, the selection of CAR-resistant populations would require multiple rounds of CAR T cell killing at high E:T ratios. Having a correct reference group and including appropriate biological replicates are also critical for these screenings, which should help exclude the experimental noise inherent in these screens. To acquire generalizable results, an ideal screening should be performed using multiple models, as well as multiple cell donors.

## 6. Conclusions and Perspectives

Clinical and preclinical studies have revealed both promises and challenges in CAR T cell therapy. While all models for CAR T cell research are generated with the scope for acquiring clinically relevant results, some are more scalable and provide more rapid readout of CAR T cell function (in vitro models, xenografts from cell lines) and others are more focused on recreating the tumor environment in actual patients (organoids, PDX, and immune-competent models) (Table 1). The success of CAR T cell preclinical development relies on a comprehensive way to utilize these models, understanding the indications of results on different biological processes of CAR T cells.

Clinical development of CAR T cells has acquired great benefit from these models. First, the understanding of biological mechanisms underlying CAR T cell activation, memory, and exhaustion has provided critical approaches to enhance the potency of CAR T cell products. Second, we are now able to recapitulate some of the main hurdles against effective CAR therapy, including antigen escape, T cell dysfunction, toxicity, and lack of trafficking. Such “sub-optimal” models are always used to screen CARs that are specifically designed to overcome certain challenges. While CAR T cell therapy against solid tumor has not yet resulted in satisfactory clinical outcomes, studies using preclinical models have revealed an essential role of the endogenous immune response mediating the antitumor effect following CAR T cell infusions, which will dictate the future endeavors to optimize CARs or develop combinational immunotherapy for solid tumor treatment.

We have to also realize that the current models discussed above are only partially fulfilling the requirement for robust CAR T cell evaluation. In particular, the complexity of human cancer is not yet completely recapitulated by these models. Consequently, CAR T cell antitumor function in preclinical models is not always correlated with clinical outcomes. Simulations of inter-cellular interactions between CAR T cells, tumor cells, and endogenous immune cells will be crucial approaches to improve model development. Dilemmas always exist between precious clinical samples and large numbers of models required for research. Therefore, tissue banks of organoids, PDX, and HM models, as well as close collaborations between research and clinical programs, will be essential for establishing more reliable models in the future.

## Figures and Tables

**Table 1:** Summary of the models for CAR T cell functional evaluation.

Model		Advantages	Limitations	Example Utilizations in CAR T Cell Research
**In vitro** ***models***	**Plate/Bead-bound**	Enable rapid readout; easy to scale up; less labor intensive	Only provide activation through CAR	Polyfunctionality [53,54,55] Biochemistry of CAR activation [59,60] CAR T cell-drug combination [60,61,62,63,64,65] CAR activation and TAA density [50,66]
	**TAA-expressing cells**		Variations from different costimulatory ligand expression	
	**Tumor-derived organoids**	Reconstitute tumor heterogeneity and TME.	CAR killing mechanism may be different from other models	Assess antigen escape [83] Discover new tumor antigens [88] Investigate interaction with TME [89,90,91]
**In vivo** ***models***	**Immune-compromised models**	Evaluation of human CAR T cells against human tumors	Difficult to study the interaction between CAR T cells and host immune response.	Refinement of CAR constructs [105,106,107,108,109,110,111] Memory of CAR T cells [42,63,112] CAR T cell trafficking [113,114,115] Adverse effects and CRS [109,116,120]
	**Implantable syngeneic tumor models**	Intact immune system	Non-human CAR T cells	Preconditioning regimens [123,124] CAR-induced host immunity [131,132,133,134] “Armored” CAR T cells [135,136,137,138,139,140] Targeting suppressive TME [141,142,143,144]
	**Genetically engineered mouse models**	Intact immune system; in situ tumor growth; testing early-stage CAR responses	Non-human CAR T cells; variations in TAA expression	Early-stage exhaustion [145,146] In vivo CAR generation [148]
	**Humanized mouse (HM) models**	Evaluating human CAR T cells in an intact immune system	Limited source for humanization; more time-consuming.	Transgenic HSCs for tumorigenesis [158] Off-target potential [161] CRS and adverse events [162,163]
